# Effect of nursing procedure-oriented comprehensive nursing on negative emotion and self-care ability of renal transplant recipients after operation

**DOI:** 10.4314/ahs.v25i1.28

**Published:** 2025-03

**Authors:** Yan Wang, Ping Ding, Qiaolan Yang, Min Xia, Na Li, Lihua Zhou

**Affiliations:** 1 School of Nursing, Anhui Medical University, Hefei, China; 2 Department of Urology Surgery, The First Affiliated Hospital of Anhui Medical University, Hefei, China; 3 Department of Infection Management, The First Affiliated Hospital of Anhui Medical University, Hefei, China

**Keywords:** Kidney transplantation, Nursing program orientation, Comprehensive nursing, Negative emotion, Self-protection ability, Nursing effect

## Abstract

**Background:**

To explore the effect of nursing process-oriented comprehensive nursing on postoperative negative emotions and self-protection ability of kidney transplant recipients.

**Methodology:**

A total of 80 patients after kidney transplantation were selected from June 2020 to May 2022 in our hospital as the research object, and were divided into control group (40 cases) and observation group (40 cases) by taking the time of carrying out nursing program-oriented comprehensive nursing in our hospital (June 2021 to May 2021, routine nursing). The emotional state [Hamilton Anxiety and Depression Scale (HAMA), (HAMD)], self-care ability and nursing satisfaction were compared between the two groups.

**Results:**

After intervention, the emotional state HAMA and HAMD scores in the observation group were lower than those in the control group, and knowledge level, self-concept, self-responsibility, self-care score and total satisfaction rate were higher than those in the control group, with statistical significance (P<0.05).

**Conclusion:**

Nursing procedure-oriented comprehensive nursing care can help renal transplant recipients relieve negative emotions quickly, increase patients' postoperative self-care ability, and increase patient satisfaction with nursing care, all of which have great significance and are well worth promoting.

## Introduction

Kidney transplantation involves replacing a diseased kidney with a healthy one from a donor, allowing the body to maintain basic metabolic function despite renal function loss in the recipientrefers to the transplantation of a healthy person's kidney into the body of a person with kidney disease and renal function loss, so as to replace the original diseased kidney and maintain the body's basic metabolic function^1^,^2^. At present, kidney transplantation is an important way to treat renal failure, which is of great significance to improve the survival rate of patients with advanced renal disease.

In terms of current medical technology, kidney transplantation is still complex and a major surgery, and some studies have found that the risk of complications after allogeneic kidney transplantation is generally high, and the occurrence of complications may have a direct impact on its overall surgical outcome, resulting in a decrease in patient survival. There are many types of post-transplantation complications, such as emotional-sleep symptom complex, pain-gastrointestinal symptom complex, and so on. Pain-gastrointestinal symptom clusters, etc., and timely symptom assessment and targeted nursing interventions can have a positive impact on improving prognosis.

In addition, it has been found that the postoperative health status of kidney transplant patients may have an impact on their quality of life and emotional state. Studies have concluded that recipients of living kidney transplantation commonly experience significant guilt towards the donor, which may increase the risk of negative emotions such as postoperative depression. Meanwhile, several studies have shown that kidney transplant patients mostly have emotional problems after surgery, and the risk of negative postoperative emotions is higher due to various factors such as environment and illness, which may have a certain impact on patient compliance and postoperative recovery outcomes. Therefore, it is important to promote the postoperative recovery of renal transplant patients by giving a high level of postoperative nursing interventions and timely adjustment to improve patients' treatment mindset.

However, in the actual diagnosis and treatment, patients after kidney transplantation are prone to negative emotions such as anxiety, depression and fear due to the influence of multiple factors, such as the source of kidney, economic load and long-term postoperative treatment, which may lead to negative emotions or behaviors of resistance to postoperative treatment, affecting the prognosis, increasing the risk of graft rejection and reducing the survival rate of patients. Timely providing effective nursing care for patients after kidney transplantation has a positive effect on improving the overall prognosis. Conventional nursing has limitations. With advancements in clinical nursing technology, nursing program-oriented nursing modes have been proposed to provide targeted nursing services for patients based on the four basic steps of nursing: evaluation, planning, execution, and evaluation. This approach aims to improve the standardization and effectiveness of nursing managementConventional nursing effect is limited, with the improvement of clinical nursing technology, some studies have put forward nursing program-oriented nursing mode, aiming to provide targeted nursing services for patients based on the four basic steps of nursing evaluation, planning, execution and evaluation, and further improve the standardization and standardization of nursing management^3^. Comprehensive nursing takes nursing procedure as the core and systematizes nursing procedure, which is conducive to giving patients more comprehensive and comprehensive nursing intervention and plays an important role in improving the overall nursing level and quality^4^. Few studies have been conducted on the application of nursing process-oriented comprehensive nursing after kidney transplantation in China. This study aims to compare the effectiveness of conventional nursing and conventional + nursing process-oriented comprehensive nursing modes in patients after kidney transplantation.

The study will investigate the impact of the two nursing modes on patients' emotional well-being and self-care ability, providing valuable insights for selecting a nursing mode after kidney transplantation At present, there are few researches on the application of nursing process-oriented comprehensive nursing after kidney transplantation in China, so this study intends to take patients after kidney transplantation as the intervention object, compare the application effect of conventional nursing and conventional + nursing process-oriented comprehensive nursing mode, in order to explore the influence of the two nursing modes on patients' emotion and self-care ability, in order to provide reference for the choice of nursing mode after kidney transplantation.

## Methods

### Patients

From June 2020 to May 2022, 80 patients treated in our hospital after kidney transplantation were selected as the research object. Inclusion criteria were as follows: 1) Patients fit for kidney transplantation^5^ Diagnosis and treatment criteria, preoperative examination meets surgical indications; 2) patients' age ≥18 years; 3) Patients receiving kidney transplantation for the first time; 4) Patients with complete general information; 5) After kidney transplantation, the patient's condition was stable without serious infection or other complications. Exclusion criteria set as follows: 1) Patients with contraindications of kidney transplantation; 2) There was coagulation dysfunction; 3) Patients had mental illness, poor compliance; 4) Patients had participted in other experiments. The time of carrying out the nursing process-oriented comprehensive nursing in our hospital (June 2021) was set as the cut-off point, they were divided into control group (40 cases, from June 2020 to May 2021, routine nursing) and observation group (40 cases, from June 2021 to May 2022, routine + nursing process-oriented comprehensive nursing mode). In control group, there were 26 males and 14 females, aged 20-60 years, average (40.03±6.29) years, waiting for kidney transplantation for 2-18 months, average (10.04±1.95) months. In observation group, there were 21 males and 19 females, aged 22 to 59 years, average (40.51±6.67) years, waiting for kidney transplantation for 3 to 18 months, average (10.52±2.01) months.

Compared with general data, there was no statistical significance (P>0.05). The patient and his family agreed and signed the informed consent, which was reviewed and approved by the The First Affiliated Hospital of Anhui Medical University hospital ethics Committee(-No.AHMU202005012).

### Nursing measures

Routine care was implemented in the control group. We strengthen the monitoring of vital signs, guide the patients to take the drug on time according to the doctor's advice, strengthen the communication with the patients, and explain the health knowledge to the patients and their families in a simple and clear way of communication. According to the patient's condition, we appropriately guide them to carry out exercise rehabilitation exercises. In the observation group, nursing procedure-oriented comprehensive nursing was implemented on the basis of routine nursing, and the nursing was as follows.

After the patient was admitted to hospital, the nurse could use one-to-one communication, in-depth investigation to understand the general information of the patient, and evaluated and analyzed the patient's current physical and mental health status. Then we combined with the basic situation of the ptient, the patient's physical and mental health factors of the secondary assessment, took the initiative to inquire, understand, analysis of the patient's anxiety and depression, unhealthy diet, sleep disorders and other issues affecting the factors.

According to the nursing problems learned during the evaluation process, combined with the basic needs of patients, we developed a comprehensive nursing plan with certain pertinence and clarified the nursing precautions.

We implemented nursing measures according to nursing plan. 1) Health education. We took the initiative to conduct in-depth communication with the patients, and asked the patients whether they encountered difficulties in a guided manner, such as “do you think that taking medicine as prescribed by the doctor has a serious impact on their own living conditions?”. We understood and evaluated the patient's perception of relevant health knowledge, carried out targeted health knowledge education for them, regularly organized the patient to carry out a health lecture, answered questions for the patient on the spot, corrected cognitive misunderstandings, guided the patient to share experiences and experiences during the lecture, and timely corrected wrong self-care behaviors. 2) Psychological nursing. Successful case telling method was adopted to explain successful medical records of similar diseases to improve patients' confidence in successful treatment. Health promotion manuals printed with Hamilton Anxiety Scale (HAMA) and Hamilton Depression Scale (HAMD) were issued to patients, and patients were instructed to master the self-assessment method of the mood Scale, and patients were told to conduct their own assessment of the Scale once a week. If negative emotions such as Anxiety and depression existed, they should seek help from professional medical personnel in time. (3) Exercise rehabilitation guidance. According to the specific conditions and needs of patients, a rehabilitation training plan was made, with yoga, tai Chi and other aerobic exercises as the appropriate, 3 times a week, 30 min each time. We assisted patients to set rehabilitation training goals, such as early training to maintain the standard of training movements and complete basic training. (4) Life diet intervention. Patients were instructed to master the self-test method of basic vital signs, and were instructed to detect and record the changes in the expression levels of basic vital signs such as blood sugar, blood pressure, heart rate and body weight every day. Self-test report forms and daily life schedule were issued, and patients were guided to complete the survey and record of the forms, and to recycle the forms regularly, so as to dynamically master their diet and body health status. Patients were instructed to adjust their lifestyle and diet, keep a light diet, low sugar, low salt and low cholesterol, and strengthen the intake of high-quality protein. Assist patients to develop good habits of going to bed early and getting up early, and tell them not to fall asleep after 11 pm, and to sleep between 10 pm and 11 pm, so as to ensure adequate body cultivation. 2 weeks after the nursing intervention, the responsible nurse instructed the patients to fill out Newcastle Satisfaction with Nursingscales (NSNS) independently to understand and summarize the nursing problems, analyzed the causes of the problems, and formulated feasible improvement countermeasures to further improve the nursing quality.

### Evaluation Methods

Relevant nursing indicators before and after intervention (2 weeks after nursing intervention) were observed and recorded.

1) Negative emotion: HAMA and HAMD scores^6^ of patients were observed and recorded, HAMA was 0∼56 points, HAMD was 0∼54 points, and the scores were negatively correlated with the improvement of mood.2) Self-care ability: Knowledge level (17-68 points), self-concept (8-32 points), self-responsibility (6-24 points) and self-care ability (14-48 points) were measured by Exercise of Self-care Agency Scale (ESCA)^7^. The scores were positively correlated with self-care ability. 3) Nursing satisfaction: the nursing satisfaction of patients was observed and recorded 2 weeks after nursing intervention according to the Newcastle satisfaction with nursing (NSNS) scales^8^. Very satisfied means that the renal function of the patient has recovered significantly, and the patient can basically develop a healthy and regular diet habit, and maintain a stable and positive treatment mentality; Satisfaction refers to the patient's general recovery of renal function, most of the bad living and eating habits have been improved, sometimes there will be significant negative emotions, but can be timely relieved; Dissatisfaction refers to poor recovery of renal function, failure to develop healthy and regular eating habits, or frequent negative emotions, affecting prognosis. Total satisfaction = very satisfaction + satisfaction.

2) The scales were used in the research were described as follows.

The HAMA is a rating scale used to measure the severity of anxiety symptoms in adults. It consists of 14 items that assess both psychological and somatic symptoms of anxiety, such as tension, fear, insomnia, and gastrointestinal symptoms. Each item is rated on a scale of 0 to 4, with a total score ranging from 0 to 56. A higher score indicates greater anxiety severity.

The HAMD is a rating scale used to measure the severity of depressive symptoms in adults. It consists of 21 items that assess both psychological and somatic symptoms of depression, such as mood, guilt, insomnia, and gastrointestinal symptoms. Each item is rated on a scale of 0 to 4, with a total score ranging from 0 to 63. A higher score indicates greater depression severity.

The ESCA scales are designed to provide a comprehensive assessment of an individual's self-care agency and can be used to help identify areas where an individual may need additional support or resources to improve their overall well-being.

The NSNS scales are designed to be used with adult patients and cover a range of nursing care areas, such as pain management, medication administration, communication, and respect for privacy. The NSNS can be used to evaluate the quality of nursing care in hospitals, clinics, and other healthcare settings. It is a widely used tool in nursing research and has been translated into several languages

### Statistical analysis

The statistical analysis was conducted using SPSS version 25.0 software (IBM Corp., Armonk, NY, USA). The continuous variables were presented as mean ± standard deviation and were tested for normality using the Shapiro-Wilk test. For inter-group comparisons of continuous variables, independent sample t-tests were used when the data were normally distributed. For categorical variables, data were presented as frequency and percentage and were compared using the *χ*2 test. A P value less than 0.05 was considered statistically significant. All tests were two-tailed. SPSS 25.0 software (IBM Corp., Armonk, NY, USA) was used for statical analysis. The measurement data were in accordance with the normal distribution represented by (x̅ ± s), the independent sample t-test was used for inter-group comparison, and the count data was represented by [cases (%)], *χ*^2^ test was conducted. P<0.05 was considered to be statistically significant.

## Results

### The negative emotions of the two groups

HAMA and HAMD scores of the observation group were lower than those of the control group after intervention, with statistical significance (P<0.05), as shown in Table 1.

**Table 1:** Comparison of negative emotions between the two groups (x±s, points)

Group	Number of cases	HAMA		HAMD	
		Pre-intervention	Post-intervention	Pre-intervention	Post-intervention
Observation group	40	27.75±4.65	12.11±2.07	26.12±3.46	10.77±2.01
Control group	40	28.07±5.01	16.85±3.06	25.96±3.38	15.85±2.52
t		0.296	8.115	0.209	9.967
P		0.768	<0.001	0.835	<0.001

### The self-care ability of the two groups

After intervention, the knowledge level, self-concept, self-responsibility and self-care score of the observation group were higher than those of the control group, with statistical significance (P<0.05), as shown in Table 2.

**Table 2:** Comparison of self-protection ability between the two groups (x±s, points)

group	Number of cases	Level of knowledge		Self concept		Self responsibility		Self-care	
		Pre-intervention	Post-intervention	Pre-intervention	Post-intervention	Pre-intervention	Post-intervention	Pre-intervention	Post-intervention
Observation group	40	30.12±4.19	46.01±5.34	20.84±3.82	26.32±3.18	13.01±2.81	21.03±3.48	24.83±3.92	34.92±3.18
Control group	40	30.94±4.64	35.83±4.82	21.38±3.77	23.01±3.02	12.94±2.78	16.18±3.19	25.01±3.88	28.72±2.11
t		0.830	8.950	0.636	4.774	0.112	6.498	0.206	10.275
P		0.409	<0.001	0.526	<0.001	0.911	<0.001	0.837	<0.001

### The nursing satisfaction between the two groups

The total satisfaction rate of the observation group was higher than that of the control group, with statistical significance (P<0.05), as shown in Table 3.

**Table 3:** Comparison of nursing satisfaction between the two groups [Example (%)]

group	Number of cases	Very satisfied	satisfactions	dissatisfaction	Total satisfaction rate
Observation group	40	24 (60.00)	16 (40.00)	0 (0)	40 (100.00)
Control group	40	13 (32.50)	23 (57.50)	4 (10.00)	36 (90.00)
*χ* ^2^					10.526
P					0.001

## Discussion

Nursing process-oriented comprehensive nursing can improve patients' emotional state.

Kidney transplantation is the most ideal treatment for bilateral renal dysfunction and chronic renal insufficiency at the end stage. It is of great significance to prolong the life expectancy and improve the survival rate of patients^6^ patients^9^. According to the different kidney sources, kidney transplantation can be generally divided into autologous kidney transplantation, allogeneic kidney transplantation and xenogeneic kidney transplantation. Due to the non-primary kidney, some patients may have different degrees of rejection or complications after kidney transplantation, such as renal function loss of the transplanted kidney and early postoperative renal insufficiency, which may directly affect the overall surgical effect, patient survival, treatment and survival rate^10^.

Maintaining a good treatment mentality and improving self-care ability play an important role in reducing the risk of adverse events after kidney transplantation. Therefore, this study applied nursing degree-oriented comprehensive nursing to the postoperative nursing of kidney transplantation recipients. Research data showed that HAMA and HAMD scores of the observation group after intervention were lower than those of the control group (P<0.05), suggesting that this nursing mode has a positive effect on improving patients' negative emotions. Analysis of the reasons are as follows: 1. Use of nursing process-oriented comprehensive nursing, before the implementation of nursing mode, through evaluation and planning, the patient's baseline information, illness, personality characteristics, psychological and emotional state of a comprehensive investigation and understanding, in-depth analysis of the patient's possible nursing problems, and develop a comprehensive nursing program with certain targeted, conducive to the responsible nurses for different patients, effective control of stimulus sources^8^ sources^11^. Effectively solve all kinds of emotional problems of different patients, guide patients from the change of subjective initiative, so that patients really realize the importance of medication according to the doctor's advice, adjust and improve bad living and eating habits. Second, through secondary evaluation, analyze and summarize the core causes of patients' negative emotions, and solve the source problems of patients' negative emotions^12^.

In order to guide patients to master the scale self-evaluation method, regular emotional state assessment, anxiety, depression, timely seek help from medical staff, to relieve anxiety, depression and other negative emotions, to help patients maintain a good and healthy emotional state, appropriate for patients to explain successful medical records, improve patients' confidence in successful treatment.

### Nursing process-oriented comprehensive nursing can improve patients' self-care ability

The data of this study showed that the knowledge level, self-concept, self-responsibility and self-care score of the observation group were higher than those of the control group after intervention (P<0.05), suggesting that nursing program-oriented comprehensive nursing has a positive impact on improving the self-care ability of renal transplant recipients after surgery. The reasons are analyzed as follows:

1. In the process of health education, responsible nurses actively guide patients to inquire whether they encounter relevant difficulties. Through the guidance of inquiry, they can deeply understand patients' cognition of their own conditions and relevant matters for attention after kidney transplantation. Organize the exchange of experience and experience among patients to further improve their cognition of relevant health knowledge.
2. By issuing self-examination report forms and daily life schedule, patients are guided to learn to regularly self-measure blood glucose, blood pressure, heart rate and body weight, understand and analyze their own health status changes, and have a more intuitive and clear cognition of the improvement of body health under doctors' advice and good nursing. At the same time, by regularly recording the changes of vital signs and the diet plan, correcting and restraining the unhealthy diet habits, forming a good and healthy diet habits, going to bed early and getting up early, reducing the kidney load, which has a positive impact on promoting the recovery of renal function, facilitating patients to feel the recovery of body health more intuitively, facilitating the formation of a virtuous cycle, further improving patients' compliance, effectively assisting patients to master self-care skills, and playing an important role in improving their overall self-care ability^10^ ability^13^,^11^,^14^.

### Nursing process-oriented comprehensive nursing can improve nursing satisfaction

The data of this study showed that the total satisfaction rate of the observation group was higher than that of the control group (P<0.05), suggesting that the nursing program-oriented comprehensive nursing plays an important role in improving patient satisfaction. The reasons are as follows:

1. Through the two steps of evaluation and planning, it is beneficial for nurses to have a better understanding of patients' conditions and needs, to provide more targeted psychological, diet and sports rehabilitation nursing, to guide patients to carry out sports and exercise suitable for their body recovery, to promote the body health recovery, and to improve the overall prognosis effect of kidney transplantation^12^ transplantation^15^,^13^,^16^. Second, based on the “evaluation” procedure to investigate and understand the nursing satisfaction of patients, timely summarize and analyze the nursing problems, and put forward targeted suggestions for improvement, to further optimize the nursing program, which plays an important role in improving the nursing satisfaction of patients. Therefore, compared with the control group, the nursing satisfaction of patients in the observation group was higher.

## Limitations

However, this study still has the following shortcomings:

1. The number of cases included in the study is limited, and there are errors in the study results;
2.2. Only two weeks after nursing nursing indicators were investigated and counted, the research time was limited, and the long-term effect and extensive effect of this nursing model could not be confirmed.

## Conclusion

The adoption of nursing program-oriented comprehensive nursing can help kidney transplant recipients timely relieve negative emotions, improve patients' postoperative self-care ability, help patients maintain a stable and positive treatment mentality, improve bad living and eating habits, and is of great significance to promote the recovery of renal function and improve patients' nursing satisfaction, which is worthy of promotion and application.

## Limitations

However, this study still has the following shortcomings:

1. The number of cases included in the study is limited, and there are errors in the study results;
2.2. Only two weeks after nursing nursing indicators were investigated and counted, the research time was limited, and the long-term effect and extensive effect of this nursing model could not be confirmed.
